# Sex-Mediated Differences in LPS Induced Alterations of TNFα, IL-10 Expression, and Prostaglandin Synthesis in Primary Astrocytes

**DOI:** 10.3390/ijms19092793

**Published:** 2018-09-17

**Authors:** Dmitry V. Chistyakov, Nadezda V. Azbukina, Alina A. Astakhova, Sergei V. Goriainov, Viktor V. Chistyakov, Marina G. Sergeeva

**Affiliations:** 1 Belozersky Institute of Physico-Chemical Biology, Moscow State University, Moscow 119992, Russia; alina.an.astakhova@gmail.com (A.A.A.); mg.sergeeva@gmail.com (M.G.S.); 2 Laboratory of electrophysiology, Pirogov Russian National Research Medical University, Moscow 117997, Russia; 3 Faculty of Bioengineering and Bioinformatics, Moscow Lomonosov State University, Moscow 119234, Russia; ridernadya@gmail.com; 4 SREC PFUR, Peoples’ Friendship University of Russia (RUDN University), Moscow 117198, Russia; goryainovs@list.ru (S.V.G.); chistvic@gmail.com (V.V.C.)

**Keywords:** sex difference, neuroinflammation, COX-2, 3β-HSD, astrocytes, trilostane, LPS, TLR4, IL-10, TNFα

## Abstract

Although many neurological and psychiatric disorders reveal clear sex-dependent variations, the molecular mechanism of this process is not clear enough. Astrocytes are involved in the response of neural tissue to injury and inflammation, produce steroid hormones, and sense steroid presence. To explore the hypothesis that astrocytes may participate in sex-mediated differences of inflammatory responses, we have examined whether male and female primary rat astrocytes show different responses to lipopolysaccharide (LPS) as a toll-like receptor 4 (TLR4) agonist. Levels of mRNA and proteins of tumor necrosis factor alpha (TNFα), interleukin-10 (IL-10), and cyclooxygenase (COX)-2 were assessed using qPCR, immunoblotting, and ELISA. UPLC-MS/MS was used to detect prostaglandins (PGs). LPS stimulation resulted in different levels of cytokine production; more TNFα and less IL-10 were produced in female cells compared with male astrocytes. Although the levels of the COX-2 expression were not altered, LPS significantly induced the synthesis of PGs with notable sex-related differences. PGE_2_ and PGD_2_ were less and 6-keto-PGF_1α_ was more upregulated in female astrocytes, and TXB_2_ had similar levels in cells obtained from males and females. Trilostane, an inhibitor of 3β-Hydroxysteroid dehydrogenase (3β-HSD), inhibited the LPS-induced TNFα production and the release of PGE_2_, PGD_2_, and 6-keto-PGF_1α_ in female astrocytes. Thus, male and female astrocytes differentially respond to inflammatory challenges on the level of production of cytokines and steroid hormones. Sex-mediated differences in pro- and anti-inflammatory responses should be taken into consideration for the effective treatment of disorders with neuroinflammation.

## 1. Introduction

Sex-dependent variation in the sensitivity of humans to some diseases has been known for a long time (see for reviews [1,2,3,4]), although, in experimental and clinical studies, the sex of subjects is still rarely taken into account. Only recently within the context of ideas of personalized medicine have researchers focused on identifying the molecular differences between men and women’s responses to external environmental pollutants, pro-inflammatory stimuli, and pharmaceuticals [5,6]. Mechanisms underlying sex-dependent response variations are of special significance for the brain, as pathologies of the central and the peripheral nervous systems demonstrate significant differences in the incidence, symptomatology, and/or neurodegenerative outcome for males and females. Examples include Parkinson’s disease, Alzheimer’s disease, Huntington’s disease, multiple sclerosis, traumatic brain injury, stroke, autism, schizophrenia, depression, anxiety disorders, eating disorders, and peripheral neuropathy [3,4,7]. Moreover, recent scientific data has provided a link between different sensitivity of males and females to organophosphate cholinesterase inhibitors, which are drugs used within the course of a therapy against Alzheimer disease, and sex-dependent differences in cholinergic system [6]. Therefore, to provide effective and safe therapy, special attention should be paid to sex-dependent variations in molecular mechanisms that provide responses and mediate the actions of various drugs in the brain.

Sex is considered among the factors that contribute to incidence and progression of diseases associated with immune responses (i.e., inflammation). It is assumed that neuroinflammation plays an important role in neurodegenerative diseases and some other brain pathologies [8,9,10,11,12], as the leading role in these processes belongs to immunocompetent cells, first of all microglia and astrocytes [12]. Sex dependence in the responses of microglia to pro-inflammatory stimuli have been well documented, although molecular mechanisms are still discussed [1,3,13,14]. Less is known about the differences in sensitivity of male and female astrocyte to pro-inflammatory substances. Sex differences in the responses of astrocytes to lipopolysaccharide (LPS) as an agonist of toll-like receptors type 4 (TLR4) were demonstrated on the mRNA level [13]. It has previously been shown that TLR4 activation in astrocytes simultaneously induced, on a protein level, both the pro-inflammatory cytokine tumor necrosis factor alpha (TNFα) and anti-inflammatory cytokine interleukin-10 (IL-10) [15,16]. The modulation of the cyclooxygenase (COX)-2 expression by LPS induces synthesis of various prostaglandins (PG) [17,18], which, modifies both the pro-inflammatory and anti-inflammatory processes, depending on the cellular context [19,20,21]. Although there is no doubt that PGs are involved the inflammatory processes that accompany many brain pathologies and steroids-prostaglandin interconnections [21], there are no data concerning sex difference in astrocyte prostaglandin synthesis during the cellular response to inflammatory stimuli. Therefore, the question arises whether astrocytes, which contribute to neuroinflammation, also contribute to the difference between males and females, through the release of inflammatory markers cytokines and prostaglandins.

An important issue of sex differences in the astrocyte responses to inflammatory challenges concerns the fact that astrocytes belong to the so-called intracrinology system [22], thus these cells express enzymes involved in steroid synthesis and metabolism, and are sensitive to their presence [10,23,24,25]. Astrocytes appear to be the most active steroidogenic cells in the brain and sense various steroids [23,26]. To evaluate the opportunity to regulate the sex-dependent differences in the LPS-induced inflammatory responses of astrocytes, we have tested whether trilostane, a competitive inhibitor of the 3β-hydroxysteroid dehydrogenase (3β-HSD, [27]), could differentially modulate the LPS-induced responses of astrocytes obtained from male and female rats. 3β-HSD is the key enzyme that catalyzes a conversion of pregnenolone, dehydroepiandrosterone (DHEA), and other precursors into pregnesolone, androstendion, and other active substances, which possess anti-inflammatory and neuroprotective properties [10,25,28,29]. We hypothesized that the astrocyte obtained from male or female brains would have different levels of cytokines and prostaglandins in conditions with or without a pro-inflammatory challenge, and trilostane would have an anti-inflammatory effect that might reveal a sex-dependent variety. To verify our assumptions, we have analyzed the mRNA and protein expression of cytokines TNFα (a pro-inflammatory cytokine) and IL-10 (an anti-inflammatory cytokine), as well as the expression of cyclooxygenase-2 (COX-2) (an enzyme with both pro-inflammatory and anti-inflammatory actions) [18,30] in cells obtained from males or females in conditions with or without LPS. We also measured the extracellular levels of PGE_2_, PGD_2_, TXB_2_, and 6-keto-PGF_1α_. The obtained results indicate that the primary astrocytes derived from male and female rat pups show a different expression of a pro-inflammatory marker TNFα and prostaglandins, as well as expression of an anti-inflammatory marker IL-10 in response to LPS. Our data implies the possibility to use trilostane as a component of an anti-inflammatory therapy in female subjects.

## 2. Results

### 2.1. Astrocytes Isolated from Male or Female Pups Reveal Similar Morphology, But Demonstrate Differences in Responses to LPS

**Figure 1:**
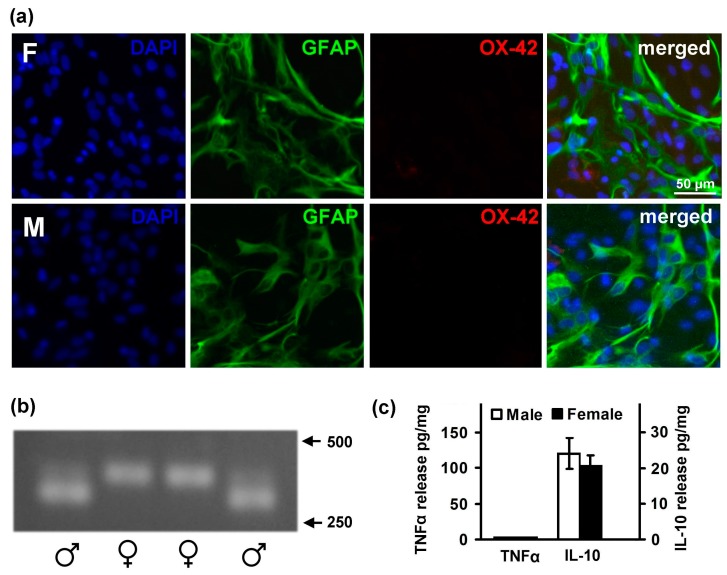
Comparison of astrocyte cultures obtained from male and female rats. (**a**) Representative immunofluorescence images showing female (F) and male (M) astrocytes cell culture morphology and purity. The cultures were fixed with 3% paraformaldehyde and incubated with DAPI (4′,6-diamidino-2-phenylindole, blue), OX-42 (Anti-CD11b/c antibody OX-42, red), and GFAP (glial fibrillary acidic protein, green). The last panels are the merged images; (**b**) An example of agarose (2%) electrophoresis of PCR products. Samples obtained from male tails have two products (margin lanes), and from the female tails—one product (central lanes). All of the products have length between 250 and 500 bp. The molecular weight marker ladder is not shown; (**c**) Comparison of basal levels of released mediators. TNFα (left scale) and IL-10 (right scale) concentrations were measured by ELISA in supernatant samples of male (white bars) and female (black bars). Values represent mean ± SEM from three independent experiments performed in triplicate.

In the first step, we evaluated the differences in the morphology of primary astrocytes derived from whole brains of male or female pups. GFAP (glial fibrillary acidic protein) was used as a marker for astrocytes, OX-42 (Anti-CD11b/c antibody OX-42) was used to detect microglia, and DAPI (4′,6-diamidino-2-phenylindole) was used to stain nuclei (Figure 1a). Astrocytes purity in our cultures exceeded 98% for the samples obtained from both male and female pups. For the sex determination, a PCR method followed by electrophoresis analysis was used (Figure 1b). The method (see details in the Section 4.3 in Materials and Methods) allows one to distinguish between sex, and for further experiments, a cell culture was attributed as male or female cells. We have found no morphological differences between the cell cultures obtained from males and females (Figure 1a).

Besides an impact on cell morphology, it has been previously reported that estrogen influences cytokine production in the brain [31]. As astrocytes are among the significant producers of cytokines in the brain [9,32], we have compared the basal levels of a pro-inflammatory mediator TNFα and an anti-inflammatory cytokine IL-10 in male and female astrocytes (Figure 1c). There were no statistically significant differences in the basal levels of these cytokines between the cells obtained from the pups of opposite sexes (Figure 1c).

**Figure 2:**
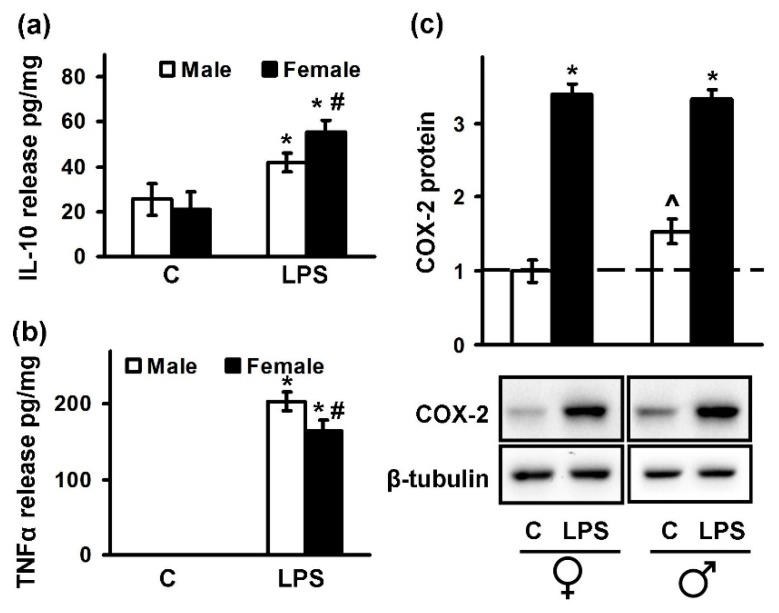
Sex differences in tumor necrosis factor alpha (TNFα), cyclooxygenase (COX)-2, and interleukin-10 (IL-10) expression during acute inflammation. Male (white) and female (black) astrocytes cultures were kept for 4 h with lipopolysaccharide (LPS) (100 ng/mL), then the concentrations of IL-10 (**a**) and TNFα (**b**) were measured by ELISA in the supernatants samples. The results are represented as mean ± SEM from three independent experiments performed in triplicate. (**c**) COX-2 protein levels were measured by Western blotting. Equal protein loading was confirmed using a β-tubulin antibody. The blot is representative of three independent experiments. * *p* < 0.05 compared with unstimulated cells, # *p* < 0.05 compared with indicated bars (sex difference), ^ *p* < 0.05 compared with male COX-2 basal protein levels.

The inflammatory responses of astrocytes to TLR agonists are well known [33,34]. It was reported that the mRNA levels of pro-inflammatory cytokines (such as IL-6, TNFα, and IL-1β) after the LPS (agonist of TLR4) treatment were higher in male than in female mice astrocytes [35]. Therefore, we decided to compare the intensity in pro- and anti-inflammatory signaling after LPS stimulation (100 ng/mL, 4 h) in male and female cultures (Figure 2). We estimated differences in the TNFα and IL-10 release levels (Figure 2a,b), as well as intracellular COX-2 protein levels (Figure 2c). We revealed that the levels of pro-inflammatory mediator TNFα were higher in the male in comparison to the female astrocytes (203 ± 12 pg/mg vs. 164 ± 15 pg/mg) (Figure 2b). In contrast, the levels of anti-inflammatory cytokine IL-10 (Figure 2a) were upregulated in female astrocytes compared with cells obtained from males. After LPS stimulation concentrations of IL-10 in media from male astrocytes were 42 ± 4 pg/mg of protein, whereas female astrocytes released 56 ± 5 pg/mg of protein.

Taken together, the set of the experiments shows that although there are no alterations in the cell morphology, the differences between males and females in responses to TLR4 activation by LPS in astrocytes do exist.

### 2.2. Sex-Mediated Differences between Male and Female LPS-Triggered Release of Prostaglandins

**Figure 3:**
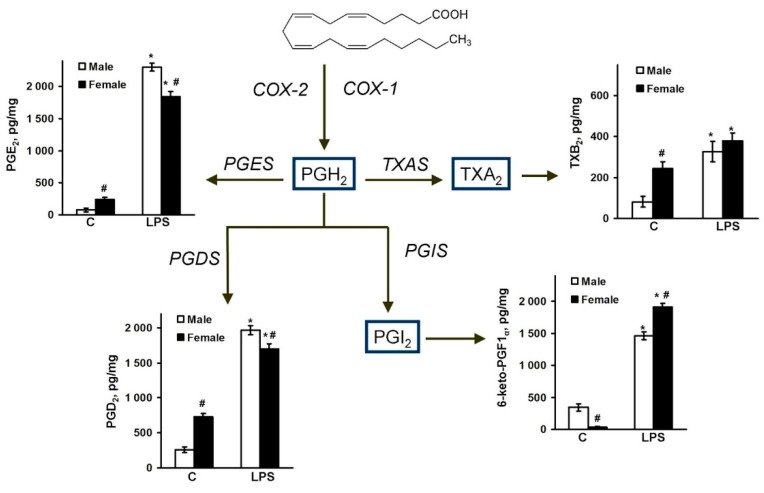
Sex differences in PGE_2_, PGD_2_, TXB_2_, and 6-keto-PGF_1α_ releases after LPS-induced inflammatory responses. Purified cultures of male and female astrocytes were treated with LPS (100 ng/mL) and concentrations of prostaglandins in culture media were analyzed by Ultra-performance liquid chromatography-tandem mass spectrometry (UPLC-MS/MS). Samples were collected 4 h after LPS. The results are represented as a scheme of the metabolic pathway with intermediate mediators (blue frame) and enzymes (above lines, representing chemical reactions). Values are represented mean ± SEM from three independent experiments performed in triplicate. * *p* < 0.05 compared with unstimaluted cells, # *p* < 0.05 compared with indicated bars (sex difference). Abbreviations: COX—cyclooxygenase; TXAS—thromboxane A synthase; PGES—prostaglandin E synthase; PGDS—prostaglandin D synthase; PGIS—prostaglandin I synthase.

Previously, it was shown that the synthesis of prostaglandins and enzymes plays an important role in the development of an inflammatory response in astrocytes [18,30,36]. Therefore, we suggested that a COX-2 activity difference was provided by a various spectrum of arachidonic acid metabolites. The cells were treated with LPS (100 ng/mL) for 4 h, and then the lipids were extracted from the cell supernatant samples using solid-phase-extraction method. Concentrations of PGE_2_, PGD_2_, 6-keto-PGF_1α_, and TXB_2_ were determined by UPLC-MS/MS, as described in the methods section. It is known that COX enzymes metabolize arachidonic acid to prostaglandins PGG_2_ and PGH_2_ (Figure 3). PGH_2_ is rapidly converted by cell-specific prostaglandin isomerases into various prostaglandins, resulting in synthesis PGE_2_ via PGES (prostaglandin E synthase), PGD_2_ via PGDS (prostaglandin D synthase), prostacyclin (PGI_2_) via PGIS (prostaglandin I synthase), and thromboxane A2 (TXA_2_) via TXAS (thromboxane A synthase) (Figure 3). TXA_2_ and PGI_2_ are unstable, therefore their production is typically monitored by the measurement of the production of stable analogs TXB_2_ and 6-keto-PGF_1α_, respectively (Figure 3).

It was found that female astrocytes produce more PGE_2_, TXB_2_, and PGD_2_, and less 6-keto-PGF_1α_ in basal conditions compared with male astrocytes (Figure 3). LPS caused a strong accumulation of all measured prostaglandins in both male and female supernatant samples; however, the release of PGE_2_ and PGD_2_ was higher in the male astrocytes. The LPS-stimulated TXB_2_ level has no statistical differences between the male and female astrocytes, while the 6-keto-PGF_1α_ level was higher in the female astrocytes (Figure 3). So, notable sex-mediated differences for prostaglandin synthesis were revealed.

### 2.3. Sex Differences in an Inflammatory Response of Astrocytes Treated with Trilostan

**Figure 4:**
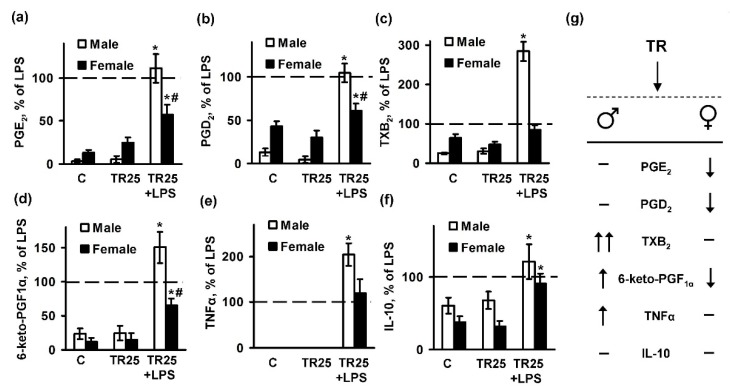
Trilostane differentially affects acute inflammatory responses in male and female astrocytes. Astrocytes were pretreated for 0.5 h with trilostane (TR; 25 μM) and then stimulated with LPS for 4 h. (**a**–**d**) Concentrations of prostaglandins in the supernatants were measured using UPLC-MS/MS; (**e**,**f**) Changes in the TNFα and IL-10 release levels in cell supernatants were measured using ELISA; (**g**) A scheme summarizing the differences in synthesis of prostaglandins, and the release of TNFα and IL-10 in male and female astrocytes upon LPS challenges (↑: increased release, ↑↑: strongly increased release, ↓: decreased release, –: no effect). All of the data are represented as ratios to LPS treatment (LPS treatment was accepted as 100%). White bars indicate the male culture, black bars indicate the female culture. Values are represented as mean ± SEM from three independent experiments performed in triplicate. * *p* < 0.05 compared with unstimulated cells, # *p* < 0.05 compared with indicated bars (sex difference).

To estimate a role of steroid hormone synthesis in the manifestation of inflammatory responses of male and female astrocytes, we exposed cells to trilostane, a competitive inhibitor of 3β-hydroxysteroid dehydrogenase. The male and female cultures were pretreated with trilostane for 30 min, and then stimulated with LPS (100 ng/mL) for 4 h. Then, the cell supernatants were collected and analyzed for cytokines and PGs production (Figure 4). Trilostane did not influence the levels of TNFα in females, but potentiated TNFα released in the male subjects (Figure 4e). The tested drug did not influence LPS-induced IL-10 release (Figure 4f). Trilostane potentiated PGD_2_ (Figure 4b) and 6-keto-PGF_1α_ (Figure 4d) release in response to LPS in male astrocyte and in contrast, reduced production of 6-keto-PGF_1α_ (Figure 4d) in female cultures. LPS-induced synthesis of PGE_2_ (Figure 4a), PGD_2_ (Figure 4b), and 6-keto-PGF_1α_ (Figure 4d) were reduced by trilostane pretreatment. Therefore, trilostane may be used for anti-inflammatory therapy in female subjects.

## 3. Discussion

An important finding in our study is that in our model of cellular inflammatory response, male and female astrocytes have no different features in their naive states, but differ in their responses to inflammatory challenges. This cellular model can be suggested for testing various substances targeting sex-mediated processes. We have shown such sex difference for trilostane, an anti-inflammatory drug with antidepressant features [37]. It is important to stress that we found no difference in pro- and anti-inflammatory marker levels in naive cells. This means that steroidogenic regulatory pathways reveal sex difference only in a course of a response to external stimuli. The significance of these finding needs to be further investigated. 

Astrocytes are important mediators of synaptic activity; neurotransmitter levels within the brain; metabolic support of neuron and participation of innate immune responses, including inflammatory reactions; and astrocytes also cross-talk with microglia by releasing inflammatory mediators. Primary astrocyte cultures present a suitable model for testing potential drugs suggested for various therapies. Our data place sex-mediated differences of inflammatory responses into the list of features that require a detailed study. It is noteworthy that, for our study, we used astrocytes derived from whole brains and this allowed us to avoid brain area-dependent variability in responses. Other models with cells obtained from different brain areas reveal difference in astrocyte morphology depending on sex [38,39]. Area-dependent differences might be explained by various astrocyte/microglia ratios. The amount of microglia varies in the different regions of the brain [24,40,41,42]. Microglia was shown to influence sex-mediated differences in responses to immune challenges [3,43], and thus interactions between microglia and astrocytes should be also taken into account [1,13]. In the present study, we obtained astrocyte cultures from whole brains, controlled microglia/astrocytes ratios, and observed no differences in the morphology and expression levels of cytokines (TNFα and IL-10) of unstimulated cells. The data with TNFα were consistent with what has been previously published [35].

In response to immune challenges with LPS, male astrocytes showed a significantly more pronounced upregulation of TNFα than female astrocytes. This observation is consistent with previous data concerning pro-inflammatory cytokine release [35]. We did not find data for IL-10 synthesis in astrocytes, but it seems rational for this anti-inflammatory cytokine to be more upregulated in female astrocytes that may be revealed in female sensitivity for diseases with inflammatory component. It is noteworthy that sex differences in the responses of astrocytes to the mitochondrial toxin 1-methyl-4-phenylpyridinium used to model symptoms of Parkinson’s disease were reported previously [44]. Most previous studies concentrated on the evaluation of proinflammatory markers. Our findings demonstrate that IL-10 upregulation also depends on sex, which might be a reason for pathologies [45]. Our present data allows one to include sex differences in future studies of solution of inflammation problem in brain. Indeed, sex-dependent differences in risk of development were described for a number of pathologies. For instance, females have a higher risk for development of multiple sclerosis and Alzheimer disease, while males have increased chances to get Parkinson’s disease and suffer from overall poorer outcomes [46,47,48]. Although astrocyte activation is associated with all CNS (central nervous system) disorders, and because many of those disorders are sexually dimorphic, little in fact is known about whether astrocyte responses for immune challenges are sex dependent and can be regulated by various substances. Our results support the idea of sex dependent responses on the level of astrocytes.

**Figure 5:**
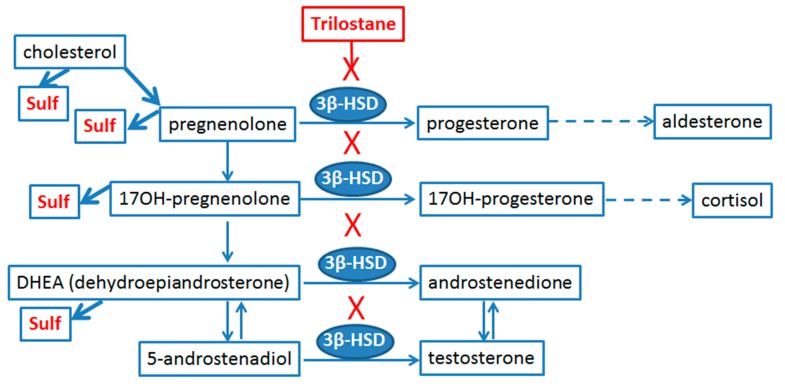
Metabolism of neuroactive steroids in astrocytes and role of trilostane as a competitive inhibitor of 3β-hydroxysteroid dehydrogenase (3β-HSD), the key enzyme of steroid transformations. “Sulf” means the sulfate metabolites of substances, “X” means the blocking effect of trilostane, straight arrows connect substrates and metabolites, and dotted arrows indicate participation of several enzymatic transformations (adapted from [49]).

Our study of trilostane does not simply validate an opportunity to use primary astrocytes as a model for testing the sex sensitivity of substances, but proves a unique role of trilostane as an inhibitor of 3β-HSD. Thus our data support opportunity to use trilostane as a substance with anti-inflammatory, antidepressant, and anxiolytic properties [37]. Indeed, steroidogenic and steroid metabolizing enzymes located in peripheral target tissues have been suggested as targets of novel therapies for steroid-sensitive diseases [22]. Astrocytes are considered to be active steroidogenic cells [23]. They produce enzymes of cholesterol and steroid metabolism (Figure 5), sensitive for the action of pregnenolone, 17-OH-pregnenolone, DHEA (dehydroepiandrosterone), progesterone, and others [10,25,28,29]. Although this has not been tested directly, it is possible to suppose that trolistane shifts the number of metabolites to the left side in reactions, presented in Figure 5. This assumption is supported by data that trilostane administration decreased the progesterone and increased pregnenolone, and increased DHEA and DHEA sulfate levels in the brain of various animals, in accordance with what could be expected for an enzymatic inhibition and substrate-to-product relationship [37,50,51].

An important finding of the present data concerns sex difference in the LPS-induced prostaglandin (PG) synthesis. Although there are still questions concerning the precise roles of various PGs in inflammation or in the resolution of inflammation, there is no doubt that signaling lipids are major regulatory participants of inflammation, developing of neurodegenerative diseases and mood disorders [16,18,30,45]. We have found only one work with correlations between PGF_2α_ and PGE_2_ synthases, TLR4 and 3β-hydroxysteroid dehydrogenase in a model, where LPS was given in vivo and then the structure and function of the bovine corpus luteum were investigated [52]. There are too few data for molecular mechanisms consideration, but an interconnection is promising. The sex difference in the basal levels of PGE_2_ and 6-keto-PGF_1α_, and the prostaglandins release under LPS stimulation were shown for human neutrophils [2]. It is noteworthy that male neutrophils have initially higher levels of both PGs, and this difference remains after LPS stimulation [2]. The molecular mechanisms and biological significance for this discrepancy remains a matter of future studies. Taken together, our present findings extend the previous observations for astrocyte sex-mediated differences in responses, and include not only cytokines, but also arachidonic acid metabolism into consideration, pointing to the importance of a consideration of sex dimorphism during the analysis of effects of these molecules and relative anti-inflammatory substances both in vitro and in vivo.

## 4. Materials and Methods

### 4.1. Reagents

LPS (Sigma-Aldrich, cat.no L2630 St. Louis, MO, USA), trilostane (cat.no SML0141, Sigma-Aldrich), streptomycin–penicillin (cat.no А063), trypsin (cat.no P037), EDTA, fetal bovine serum (cat.no BS-110/500), and the culture medium Dulbecco’s Modified Eagle Medium (DMEM) (cat.no c425) were from PanEco (Moscow, Russia). Antibodies against COX-2 (Cell Signaling Technology, D5H5, cat.no 12282, Danvers, MA, USA) and β-tubulin (Sigma Chemicals, Taufkirchen, Germany), secondary horseradish peroxidase conjugated antibodies (anti-rabbit, anti-mouse, and anti-goat) (SCBT and CST), Western Blotting Substrate ECL (Thermo Fisher Scientific, cat.no 32209, Waltham, MA, USA), and ELISA kits for TNFα (cat.no. KRC3012) and IL-10 (cat.no. BMS629) (InvivoGen, San Diego, CA, USA) were also used. The confocal antibodies OX-42 cat.no CBL1512 (1:100) and GFAP cat.no. AB5804 (1:2000) were from (Merck, Darmstadt Germany), and the secondary antibodies, anti-rabbit Alexa Fluor 488 cat.no. 111-545-003 and anti-chicken Alexa Fluor 594 cat.no. 115-585-062, were from Jackson ImmunoResearch Europe Ltd. (Suffolk, UK). The eicosanoid standards were as follows: TXB_2_-d4 (cat.no. 319030), 6-keto-PGF_1α_-d4 (cat.no. 315210), PGA_2_-d4 (cat.no. 310210), PGE_2_-d4 (cat.no. 314010), and PGD_2_-d4 (cat.no. 312010) (Cayman Chemical, Ann Arbor, MI, USA). Oasis^®^ HLB cartridge (60 mg, 3cc, cat.no. WAT094226) were obtained from Waters, Eschborn, Germany.

### 4.2. Primary Cell Culture

The cells were obtained from one or two day old pups of Wistar rats. All of the experimental procedures were performed according to the guidelines in the European Convention for the Protection of Vertebrate Animals used for Experimental and Other Scientific Purposes, and were approved by the Bioethics Committee (Protocol 2/13 from 8 April 2013) of The Department of Biology at Moscow State University. The materials from each of the pups were used for the astrocytes preparation and sex genotyping (Materials and Methods Section 4.3). The cultures of primary rat astrocytes were obtained from newborn rats of both sexes, as previously reported [53]. In brief, the brains from decapitated pups were rinsed with ice-cold Puck’s solution (137.0 mM NaCl, 5.4 mM KCl, 0.44 mM KH_2_PO_4_, 0.3 mM Na_2_HPO_4_, and 5.5 mM glucose, pH 7.4) and triturated against nylon meshes with the pores of 250 and 136 μm, in a consecutive order. The dissociated cells were plated into 75 cm^2^ culture flasks at a density of 6 × 10^5^ cells per mL. The cells were subsequently cultured in DMEM (1 g/L d-glucose, 10% bovine fetal serum [FBS], 50 units/mL streptomycin, 50 μg/mL penicillin) at 37 °C, with 10% CO_2_. After five days of cultivation in DMEM, the culture medium was replaced with fresh a medium and the flasks were placed on a shaker at 200 rpm for 4 h to dissociate the microglial cells. The microglia containing medium was discarded and the astrocytes-enriched cultures were further grown for the following four days, and the medium was replaced every two days. Subsequently, the cells were washed with phosphate buffered saline and detached from the plastic with trypsin–EGTA solution and plated into six-well plates, and were maintained for two days in DMEM. After this, the medium was replaced by the medium of the same composition, and the cells were used for the experiments. The stimulation with LPS was carried out in male and female astrocytes (100 ng/mL, 4 h). The LPS dosage was selected based on our previous studies [53,54]. In preliminary studies, all of the tested substances were estimated for toxicity by MTT (3-[4-dimethylthiazol-2-yl]-2,5-diphenyltetrazolium bromide) assay. All of the tested substances were not toxic (not shown).

### 4.3. Rat Genotyping

The DNA extraction from the tails was performed for genotyping, according to protocol described in the literature [55]. Briefly, the last 2 mm of the tails were placed into 75 μL alkaline lysis buffer (NaOH 25 mM, Na_2_-EDTA 0.2 mM) in a PCR tube. Then, the samples were heated at 95 °C for 20 min. After heating, the samples were cooled at 4 °C, and 75 μL of a neutralization buffer (Tris-HCl 40 mM) was added to each sample. The DNA concentrations were measured using IMPLEN NanoPhotometr N60. Then, 5 μL of the final preparation was used per each PCR reaction. The PCR reactions were performed on the DTlite 4 (DNA-technology, Moscow, Russia) using HS-PCR Mix (Evrogen, Moscow, Russia). The sequences of the PCR primers used in the present study were as follows: sense 5′-CTGAAGCTTTTGGCTTTGAG-3′; antisense 5′-CCACTGCCAAATTCTTTGG-3′. The sex determinations were performed using 2% agarose electrophoresis of PCR products.

### 4.4. Western Blot Analysis

The astrocytes were lysed in a modified radio immuno-precipitation assay (RIPA) buffer (50 mM Tris, pH 7.4, 1% NP-40 Sigma Chemicals, 0.25% Na-deoxycholate, 150 mM NaCl, 1 mM EDTA, 1 mM Na3VO4, 1 mM NaF) and protease inhibitor cocktail (Roche Molecular Biochemicals, Mannheim, Germany). The protein concentration was determined by the standard Bradford assay. Samples containing 20 μg of protein in a conventional Laemmli buffer were loaded on each lane of a 10% sodium dodecyl sulfate-polyacrylamide gel and subjected to a standard SDS-PAGE. After electrophoresis, the proteins were transferred onto the nitrocellulose membrane with 0.2 μm pores. The membranes were blocked in a 10% Rotiblock (Roth, Nürnberg, Germany) solution for 1 h and subsequently subjected to Phosphate-Buffered Saline with Tween 20 0.05%, with a respective primary antibody—anti-COX-2 (1:2000) at 4 °C overnight. Secondary species-specific antibodies (Dianova, Hamburg, Germany) were applied at the concentration of 1:10,000 for 1 h at room temperature. The conjugates were visualized using SuperSignal™ West Femta Chemiluminescent Substrate (Thermo Scientific). For the β-tubulin analysis, the membranes were stripped at 21 °C for 20 min with Restore Western Blot Stripping Buffer (Pierce, Bonn, Germany). The membranes were re-probed with an antibody against β-tubulin (1:10.000) from Sigma Chemicals, and secondary anti-mouse IgG (Dianova, Hamburg, Germany), to control for protein loading. The protein bands were visualized by SuperSignal™ West Pico Chemiluminescent Substrate (Thermo Scientific). Densitometry was carried out on four different experiments. The band intensity was measured using a GS-800 calibrated densitometer signal and Quantity One software (Bio-Rad, Hercules, CA, USA), and normalized to the intensity of the respective bands obtained for β-tubulin.

### 4.5. Immunofluorescence Analysis

The astrocytes were plated onto glass-bottom Petri dishes at the quantity of 10^5^ cells/glass and allowed to attach for 12 h. After the media change, the cells were left for an additional 24 h and used in the experiments, as described elsewhere. The slides with cells fixed in 3% paraformaldehyde buffered with PBS and were treated with Triton X-100 containing buffer, and were blocked with FBS and subsequently incubated overnight with primary antibodies against OX-42 (1:100) and GFAP (1:2000). The Alexa secondary antibodies, from goat, were used at the following dilutions: Alexa 488 anti-rabbit 1:1000 and Alexa 633 anti-chicken 1:1000. The images were obtained with an Axiovert 100M (Zeiss, Göttingen, Germany), equipped with confocal microscopy software LSM 510.

### 4.6. UPLC-MS/MS Conditions and Sample Preparation

After the experiments, the supernatant was collected and stored at −70 °C for further analysis. The cell-free culture media were taken for solid-phase lipid extraction (Oasis^®^ HLB cartridge [60 mg, 3cc]). For the solid-phase-extraction, 1 mL of the HLB cartridges were washed with 1 mL of methanol and 1 mL of 0.1% formic acid. A half mL of the prepared sample was loaded onto the column and washed with 1 mL 0.1% formic acid and 1 mL 15% methanol. The cartridges were then eluted with 300 μL of methanol. The lipid mediators were analyzed by 8040 series UPLC-MS/MS (Shimadzu, Kyoto, Japan), with all of specifications set as previously reported [20]. The quantification and qualification were accomplished in multiple-reaction monitoring mode, and the MS was operated at a unit mass resolution for both the precursor and product ions. The Lipid Mediator Version 2 software package was used to operate the mass spectrometer (Shimadzu, Japan). The mediators were separated based on their chemical properties in UPLC, then, we monitored their ion fragments by collision-induced dissociation in conjunction with electrospray ionization-MS/MS. TXB_2_, 6-keto-PGF_1α_, PGE_2_, PGD_2_, and PGA_2_ were identified according to accurate *m*/*z*, retention time, relative retention time of species in the same class, and the spectra of MS/MS. For the quantitative analysis of eicosanoids, all of the samples were examined by LC-MS/MS to measure the peak areas of the detected species. In order to compensate for the fluctuations in MS intensities during different runs, the peak areas of each individual lipid species were corrected by deuterated internal standards. The concentration of prostaglandins was normalized to the total protein and was expressed as pg/mg. The total protein was determined by the Bradford assay.

### 4.7. Determination of TNFα and IL-10 by Enzyme-Linked Immunoassay

After the experiments, the supernatant was collected and stored at −70 °C for the further analysis. The levels of the released TNFα and IL-10 were determined using an enzyme-linked immunoassay commercial kits and Synergy H4 plate reader (BioTek, Winooski, VT, USA), following the manufacturer’s instructions.

### 4.8. Experimental Data Analysis and Statistics

The data are expressed as mean ± SEM. The data were subjected to a one-way ANOVA, followed by Bonferroni’s post hoc test, in order to determine the statistical significance. *p* < 0.05 was considered statistically significant. All of the experiments were repeated at least three times. 

## Abbreviations

TLR	toll-like receptor
LPS	lipopolysaccharide
TNFα	tumor necrosis factor alpha
IL-10	interleukin-10
TR	trilostane
COX-2	cyclooxygenase-2
3β-HSD	3β-Hydroxysteroid dehydrogenase
